# Role of the polyamine transporter PotABCD during biofilm formation by *Streptococcus pneumoniae*

**DOI:** 10.1371/journal.pone.0307573

**Published:** 2024-08-07

**Authors:** Brenda Vieira, Jessica B. Alcantara, Giulia Destro, Maria E. S. Guerra, Sheila Oliveira, Carolina A. Lima, Giovanna B. Longato, Anders P. Hakansson, Luciana C. Leite, Michelle Darrieux, Thiago R. Converso

**Affiliations:** 1 Laboratório de Biologia Molecular de Microrganismos, Universidade São Francisco, Bragança Paulista, Brazil; 2 Laboratório de Farmacologia Molecular e Compostos Bioativos, Universidade São Francisco, Bragança Paulista, Brazil; 3 Division of Experimental Infection Medicine, Department of Translational Medicine, Lund University, Lund, Sweden; 4 Laboratório de Desenvolvimento de Vacinas, Instituto Butantan, São Paulo, Brazil; Lady Hardinge Medical College, INDIA

## Abstract

*Streptococcus pneumoniae* is a bacterium of great global importance, responsible for more than one million deaths per year. This bacterium is commonly acquired in the first years of life and colonizes the upper respiratory tract asymptomatically by forming biofilms that persist for extended times in the nasopharynx. However, under conditions that alter the bacterial environment, such as viral infections, pneumococci can escape from the biofilm and invade other niches, causing local and systemic disease of varying severity. The polyamine transporter PotABCD is required for optimal survival of the organism in the host. Immunization of mice with recombinant PotD can reduce subsequent bacterial colonization. PotD has also been suggested to be involved in pneumococcal biofilm development. Therefore, in this study we aimed to elucidate the role of PotABCD and polyamines in pneumococcal biofilm formation. First, the formation of biofilms was evaluated in the presence of exogenous polyamines–the substrate transported by PotABCD–added to culture medium. Next, a *potABCD*-negative strain was used to determine biofilm formation in different model systems using diverse levels of complexity from abiotic surface to cell substrate to *in vivo* animal models and was compared with its wild-type strain. The results showed that adding more polyamines to the medium stimulated biofilm formation, suggesting a direct correlation between polyamines and biofilm formation. Also, deletion of *potABCD* operon impaired biofilm formation in all models tested. Interestingly, more differences between wild-type and mutant strains were observed in the more complex model, which emphasizes the significance of employing more physiological models in studying biofilm formation.

## Introduction

*Streptococcus pneumoniae*, commonly known as the pneumococcus, is a Gram-positive, facultative anaerobic bacterium that may or may not be encapsulated. It is typically acquired during early childhood and most often colonizes the nasopharynx and oropharynx asymptomatically as biofilms [1, 2]. However, under certain conditions, often associated with viral infections, pneumococci can escape from the biofilm and disseminate to other niches, such as the lungs and internal organs, causing pneumonia, meningitis, and sepsis [3, 4]. Invasive infection arising from bacterial invasion is responsible for more than one million deaths per year [1, 2].

The initial and obligatory events required for colonization of *Streptococcus pneumoniae* occurs in the nasopharynx [5–7]. In this niche, the pneumococci can be found organized as biofilms, highly structured communities that are more resistant to antimicrobial agents when compared to planktonic cells grown statically in culture medium. Pneumococci grown in biofilms have a lower invasive capacity, and the biofilm provides a favorable environment for bacterial multiplication and greater exchange of genetic materials [7]. Bacterial aggregation and biofilms have also been found on mucosal surfaces during middle ear infection, sinusitis, and pneumonia [5, 8].

Many bacterial factors are important for the ability of various organisms to colonize the host. It is known that polyamines are molecules essential for growth and survival of all cells; putrescine, spermidine, spermine and cadaverine are examples of the most common polyamines and are linked to several physiological processes, such as interaction with nucleic acids, modulation of the function of RNA, DNA and nucleotide triphosphatases, and synthesis of proteins and related substances [9]. Most bacteria can synthesize polyamines, but can also transport them from the extracellular to the intracellular environment using a set of membrane proteins [10]. Furthermore, a study conducted on an abiotic surface demonstrated that the absence of polyamine transport proteins has a negative impact on the biofilm formation by an encapsulated pneumococci [11].

In *S*. *pneumoniae*, the polyamine transporter, Pot, stands out as an important ABC transporter, being responsible to transport polyamines from environment to the intracellular environment, this known as PotABCD, an ABC transporter expressed in virtually all pneumococcal strains [11–13]. Within the complex, the transmembrane substrate-binding protein PotD is responsible for capturing the polyamines from the extracellular environment. Considering its extracellular location, PotD has been investigated as a vaccine candidate against pneumococcal infection in different models [14–18]. Mice immunized with recombinant PotD showed reduced nasopharyngeal colonization by the pneumococcus and were protected against invasive challenge [14, 18, 19].

Given the importance of PotD in reducing nasopharyngeal colonization, this work aims to investigate the relationship between the polyamines and polyamine transporter PotABCD and biofilm formation by *Streptococcus pneumoniae*. We hypothesize that the content of polyamines on the bacterial cell would affect the biofilm formation by the bacterium. Our results support this hypothesis as i) our *in vitro* and *in vivo* experiments show that addition of extracellular polyamines increased biofilm formation and ii) the mutant (unable to express PotABCD) produced less biofilm than the wild type strain since it cannot import polyamines from the culture medium.

## Materials and methods

### 1. Strains and storage conditions

All *Streptococcus pneumoniae* strains used in this study are listed in Table 1. A TIGR4 isogenic mutant, ΔpotABCD, whose genes *potA*, *potB*, *potC* and *potD* (corresponding to the entire *pot* operon) were deleted was made by allelic replacement of genes belonging to the *potABCD* operon with a kanamycin resistance cassette, flanked by the upstream and downstream regions of the operon. This strain was produced and provided by Dr. Swiatlo, VA Medical Center, Section for Infectious Disease, Mississippi, USA [11]. All strains were cultivated to an O.D. _600nm_ of 0.4–0.5 in Trypticase Soy broth (TSB medium) (Kasvi) under micro-aerobic condition (5% CO_2_). Frozen stocks were produced by centrifuging bacterial culture and resuspending it at 10% of the original volume in TSB with addition of 20% glycerol and stored at -80°C until use.

**Table 1:** *Streptococcus pneumoniae* strains used in this work.

Strain	Serotype	Source	Isolation site	Reference
**0603**	6B	BCH^1^	Unknown	[20]
**245/00**	14	IAL^2^	Blood (pneumonia)	[21]
**D39**	2	UAB^3^	Unknown	[22]
**A66. 1**	3	UAB^3^	Blood	[22]
**TIGR4**	4	UAB^3^	Blood (pneumonia)	[23]
**P1079**	1	UFG^4^	Blood (pneumonia)	[24]
**P1153**	9V	UFG^4^	Blood (pneumonia)	[24]
*ΔpotABCD*	4	VA^5^	-	[25]

BCH1—Boston Children’s Hospital, USA.

IAL^2^—Instituto Adolfo Lutz, Brazil.

UAB^3^—University of Alabama at Birmingham, USA.

UFG^4^—Universidade Federal de Goiás, Brazil.

VA^5^—VA Medical Center, Section for Infectious Disease, Mississippi, USA.

### 2. Comparison of *in vitro* biofilm production by wild-type pneumococci and pot-negative mutants with and without addition of polyamines

The pneumococcal strains were first cultured to an O.D._600 nm_ of 0.3 in TSB medium under anaerobic conditions at 37°C and the cells were then transferred to 24-well plates (Corning). In each well, 10^5^ CFU of bacteria were added in 1 ml of TSB medium supplemented with 10% equine serum (Sigma-Aldrich) and incubated for 24 hours at 37°C under micro-aerobic conditions (5% CO_2_). Each strain was added to 10 wells and 4 wells were filled with medium without bacteria present, these 4 wells were considered the negative control [25, 26].

After 24 hours of incubation, the medium was discarded, the wells were washed with PBS once, and dried at room temperature. Adhered cells were stained with 0.1% crystal violet (Sigma-Aldrich) in distilled water for 30 minutes. Then, the dye was discarded, and the cells were washed again three times with PBS. The crystal violet that adhered to the biofilm was solubilized in 1 ml of 95% ethanol and incubated at room temperature for 15 min at 80 rpm agitation to solubilize the bacterial cells. The absorbance of the samples was measured in a spectrophotometer at 590 nm. The mean of the negative controls was considered as blank, and this value was subtracted from the measurement of each well filled with bacteria.

For CFU counting, the 24-well plate (Corning) was prepared as above, with each strain added to 4 wells and 2 wells filled with medium alone (negative control). Bacteria were incubated as above for 24 hours, at 37°C under micro-aerobic conditions (5% CO_2_) and the wells were washed with PBS to remove planktonic cells. Fresh PBS (500 μl) was added to each well and the biofilm was scraped off using a 20–200 μl pipette tip to release all biofilm cells. The cells were collected, diluted, and plated on blood agar plates (Probac) overnight at 37°C under micro-aerobic conditions (5% CO_2_). The following day, colonies on the plates were counted.

To determine biofilm formation in the presence of polyamines, 2 mM, 1 mM, 0.5 mM or 0.25 mM of either spermidine (Sigma-Aldrich) or putrescine (Sigma-Aldrich) were added to the medium (TSB medium supplemented with 10% equine serum). The plate was prepared using 4 wells for each condition, and 2 wells filled with medium alone (negative control), incubation used the same parameters of 24 hours, at 37°C under micro-aerobic conditions (5% CO_2_) After 24 hours the plate was washed and stained with crystal violet as described above.

### 3. Biofilm formation on cell substrate

The biofilm evaluation on cell substrate was analyzed as described by Marks et al. [6] with modification. Briefly, human lung epithelial carcinoma cells (A549 cells, ATCC CCL-185), were cultured in DMEM medium (Sigma-Aldrich) with 10% fetal bovine serum (Sigma-Aldrich) at 37°C until confluence and fixed with 4% paraformaldehyde (Sigma-Aldrich) in 24-well flat-bottom polypropylene plates. The cells were used as a substrate for the bacteria to form biofilm. For that, bacterial stocks were thawed and diluted to a 1:1,000 in TSB, 500 μl of the diluted bacteria were seeded in each well (10 wells per strains and 4 wells with just medium representing negative controls) and incubated at the nasopharyngeal temperature of 34°C in 5% CO_2_. For optimal biofilm formation the medium was changed every 12 hours, and the biofilms were left for 60 hours when the medium was discarded, and the wells washed with PBS to remove planktonic cells. Fresh PBS (500 μl) was added to each well and the biofilm was scraped off using a 20–200 μl pipette tip to release all biofilm cells. The cells were collected, diluted, and plated on blood agar plates overnight at 37°C under micro-aerobic conditions. The following day, colonies on the plates were counted.

### 4. Biofilm formation and colonization *in vivo*

The experiments described in this work were approved by the ethics committee at São Francisco University, Bragança Paulista, Brazil (CIAEP/CONCEA N° 01.226.2014, approved in March 2021). The animals were purchased from Centro Multidisciplinar Para Investigação Biológica (CEMIB–Universidade Estadual de Campinas (Campinas, Brazil).

The formula used to calculate the number of animals is as follows:

n = 1 + [2xCx(s/d)^2^], where

C is based on a Standard Normal Probability Table, considering a maximum standard deviation of 20% and an acceptable difference between groups of 31% (based on the group previous experience), with a test power of 90% and a significance level of 0.05 (recommended for this kind of experiments).

Therefore: n = 1 + [2x 10.51 x (0.2/0.31)^2^]

Resulting in n = 9.75 animals, which rounded to the nearest whole number becomes 10 animals per group.

Ten female C57Bl/6 mice (5–7 weeks) were infected as described by Converso *et al*. [27]. Briefly, wild-type and mutant strains were thawed and diluted to a concentration of 1 x 10^9^ CFU/ml in sterile PBS and each un-anesthetized animal received 10 μL (1 x 10^7^ CFUs) in one nostril/nare, the animals were checked twice a day for nine days for signs of illness and no disease was observed.

The biofilm analysis was performed as described by Marks at al. [6]. Nine days post infection, the animals were euthanized through the i.p. route with 200 μL of a mixture of 6.25 μg xylazine and 10 μg ketamine, death was confirmed by heart punction, the trachea was exposed and sectioned, and the upper respiratory tract was washed to remove planktonic bacteria. Next, the Nasal Associated Lymphoid Tissue (NALT) was removed and macerated in a homogenizing bag containing 600 μL of PBS. This material was diluted and plated on blood agar plates containing 2.5 μg/mL of gentamicin; the plates were incubated for a period of 18 h in micro-aerobic condition at 37°C for CFU counting, the bacterial load collected from each animal was plated individually generating one data point.

### 5. Statistical analysis

All statistical analysis were performed by the software GraphPad Prism 8.0. Student’s t-test was used for comparison between wild-type and mutant strains in the biofilm experiments and when only two groups were analyzed. One way ANOVA with a Dunnet’s multiple comparison post-test was used for comparison between all groups in the polyamine addition experiments, in this case the group with no polyamine supplemented was considered as reference.

For all experiments, the values were analyzed by the outlier calculator tool and the outliers were excluded from the analysis; The tool is available on GraphPad webpage (https://www.graphpad.com/quickcalcs/grubbs1/).

## Results

### 1. Biofilm formation on abiotic surface

**Fig 1:**
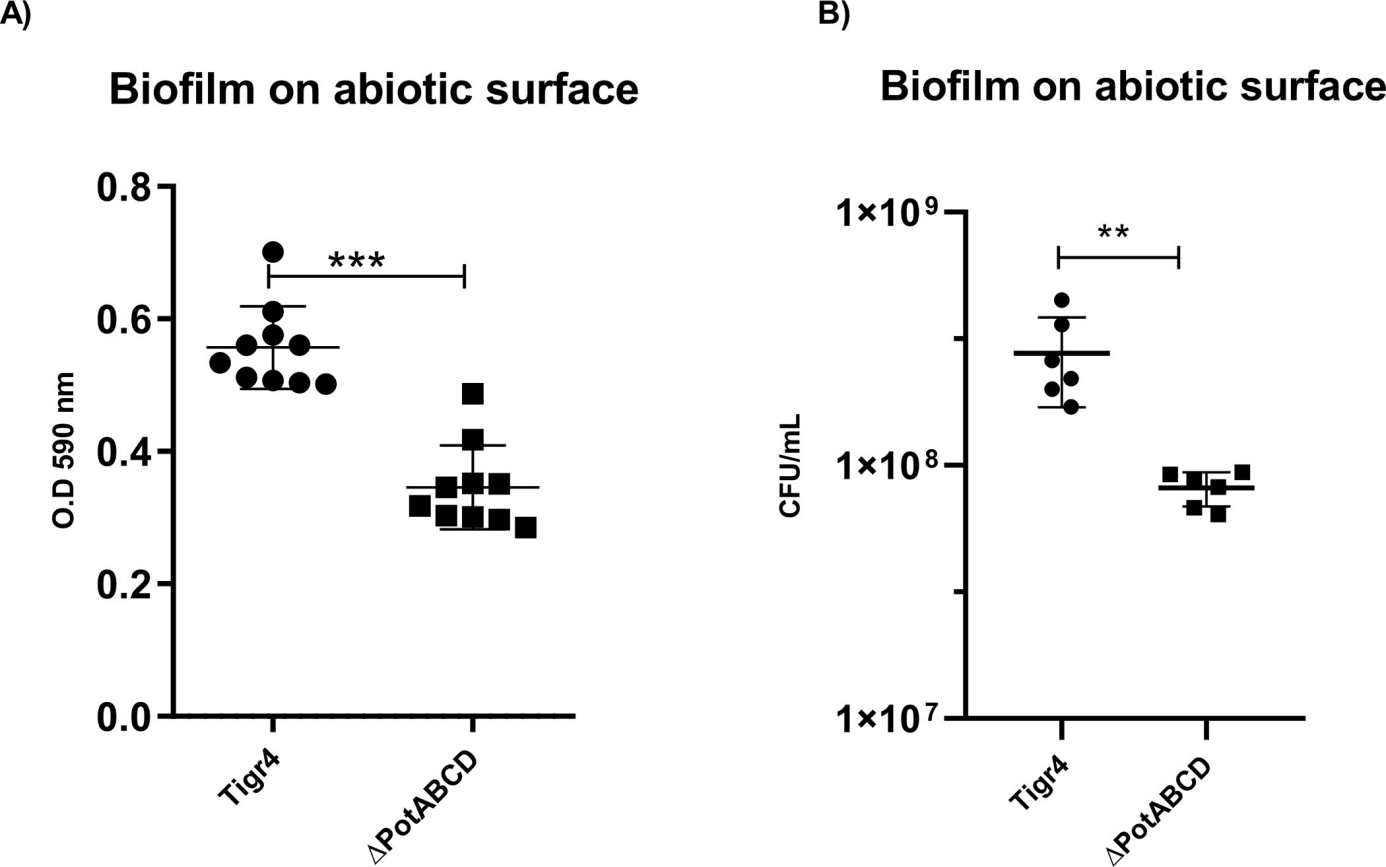
Biofilm formation on abiotic surface. The biofilm formation was evaluated after 24 h of culture in a microplate containing TSB medium supplemented with 10% equine serum. **A)** The biofilm biomass was evaluated by crystal violet staining and plate reader at 590 nm. **B)** The biofilm was evaluated by CFU counting. The results expressed are representative of three independent experiments and the difference was determined by Student’s t-test where ** = p<0.01 and *** = p<0.001.

To compare the ability of TIGR4 wild-type and ΔpotABCD strains to form biofilm, the two strains were added to a polystyrene 24-well plate and the biofilm was allowed to develop for 24 h. Fig 1A presents the difference between the strains, showing that the mutant forms almost 40% less biofilm than the wild-type bacterium. In Fig 1B, the same assay was performed but the biofilm was evaluated by CFU counts. The mutant produced 3 times less biofilm than the wild-type strain confirming the result obtained by the CV staining (Fig 1A).

### 2. Biofilm formation on cell substrate

**Fig 2:**
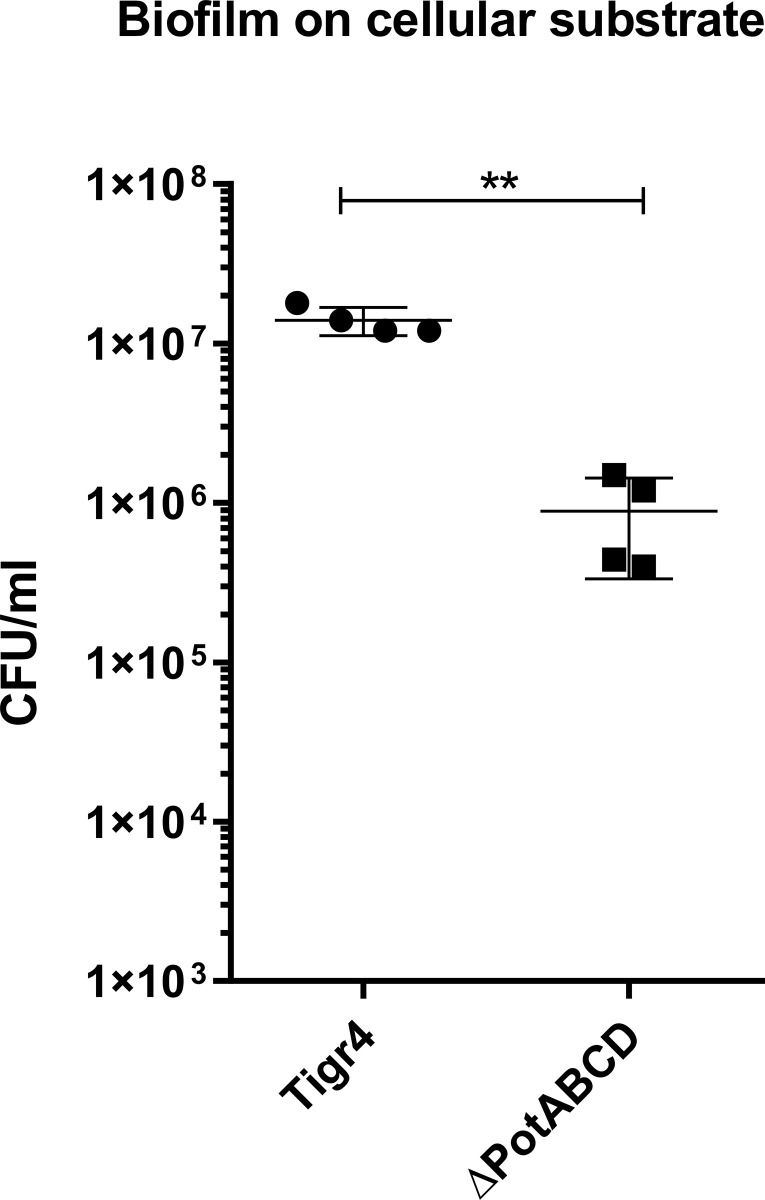
Biofilm formation on cell substrate. *S*. *pneumoniae* wild-type or ΔpotABCD strains were added to 24-well plates coated with A549 fixed cells, the medium was changed every 12 hours and bacteria were kept for 60 hours for biofilm formation. The biofilm was analyzed by plating serial dilutions on blood agar plates. The results expressed are representative of three independent experiments. The difference was determined by Student’s t test where ** = p<0.01.

The A549 cellular substrate was used to more closely mimic the human respiratory environment, the natural pneumococcal colonization niche, a protocol adapted from Chao et al. [28] to compare the biofilm formation between wild-type and *potABCD*-negative bacteria (Fig 2). The result is presented as the number of recovered CFUs and shows that the wild-type strain produces 10 times more biofilm than the mutant, reinforcing our hypothesis that polyamine transporters are important for biofilm formation even in a more physiological model, simulating the natural niche where the bacteria reside.

### 3. Polyamine transporter is important for colonization and biofilm formation *in vivo*

**Fig 3:**
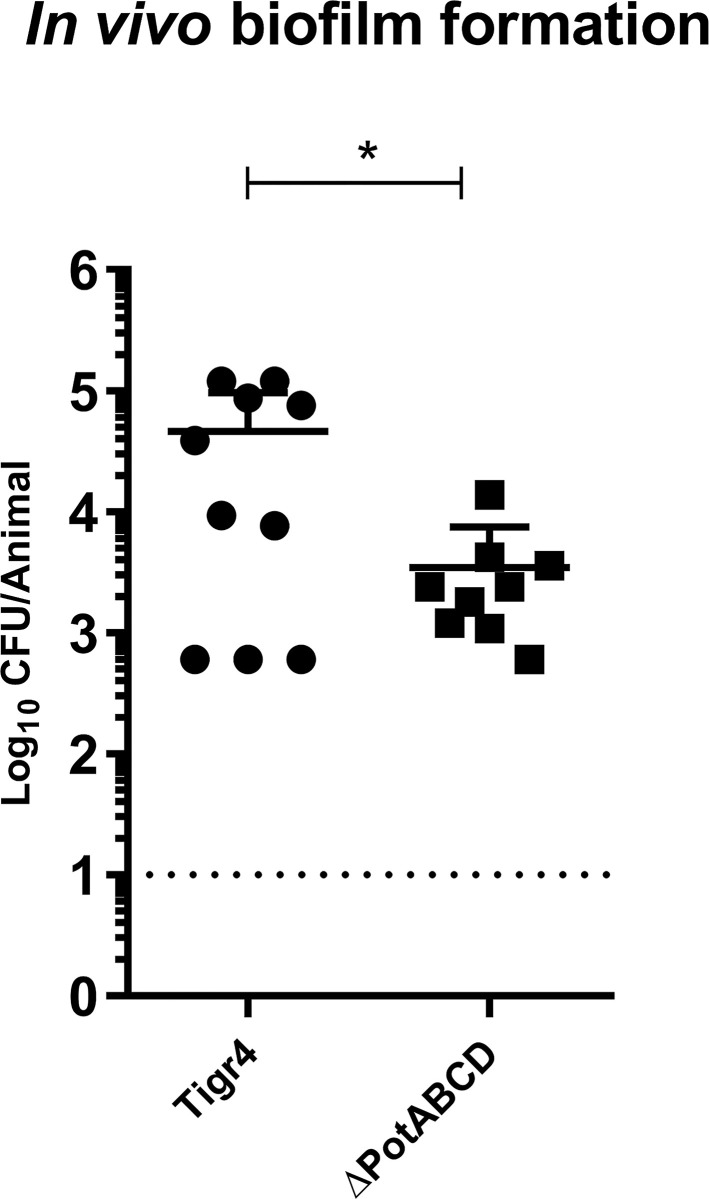
The importance of polyamine transporter on biofilm formation in vivo. C57Bl/6 mice were infected via intranasal route with 1 x 10^7^ CFU of TIGR4 or ΔPotABCD strains. On the ninth day post-infection, the Nasal Associated Lymphoid Tissue (NALT) was collected, homogenized, and plated on blood agar plates for CFU counting in each group. Each dot in the graph correspond to the number of bacteria recovered in one animal. The dotted line corresponds to the detection limit for this experiment which is 10. This graph is representative of two independent experiments. Difference was analyzed by Student t test where * = p <0.05.

To determine whether the polyamine transporter is important for biofilm formation *in vivo*, we infected C57Bl/6 animals with the wild-type or the mutant strain and allowed the bacterium to colonize and form biofilm for 9 days. At this time, the Nasal Associated Lymphoid Tissue (NALT) was collected and the recovered CFUs were counted (Fig 3). Similar to that observed on the cell substrate biofilm, the mutant bacterium that was unable to capture polyamines from the environment colonized the nasopharynx to a lower degree than PotABCD-positive wild-type bacteria counterpart. The CFUs recovered in mice infected with the wild-type strain showed more than 100 times higher bacterial load in the nasopharynx than the group infected with the mutant strain (39,000 vs 360).

### 4. Exogenous polyamines improve biofilm formation in *S*. *pneumoniae*

**Fig 4:**
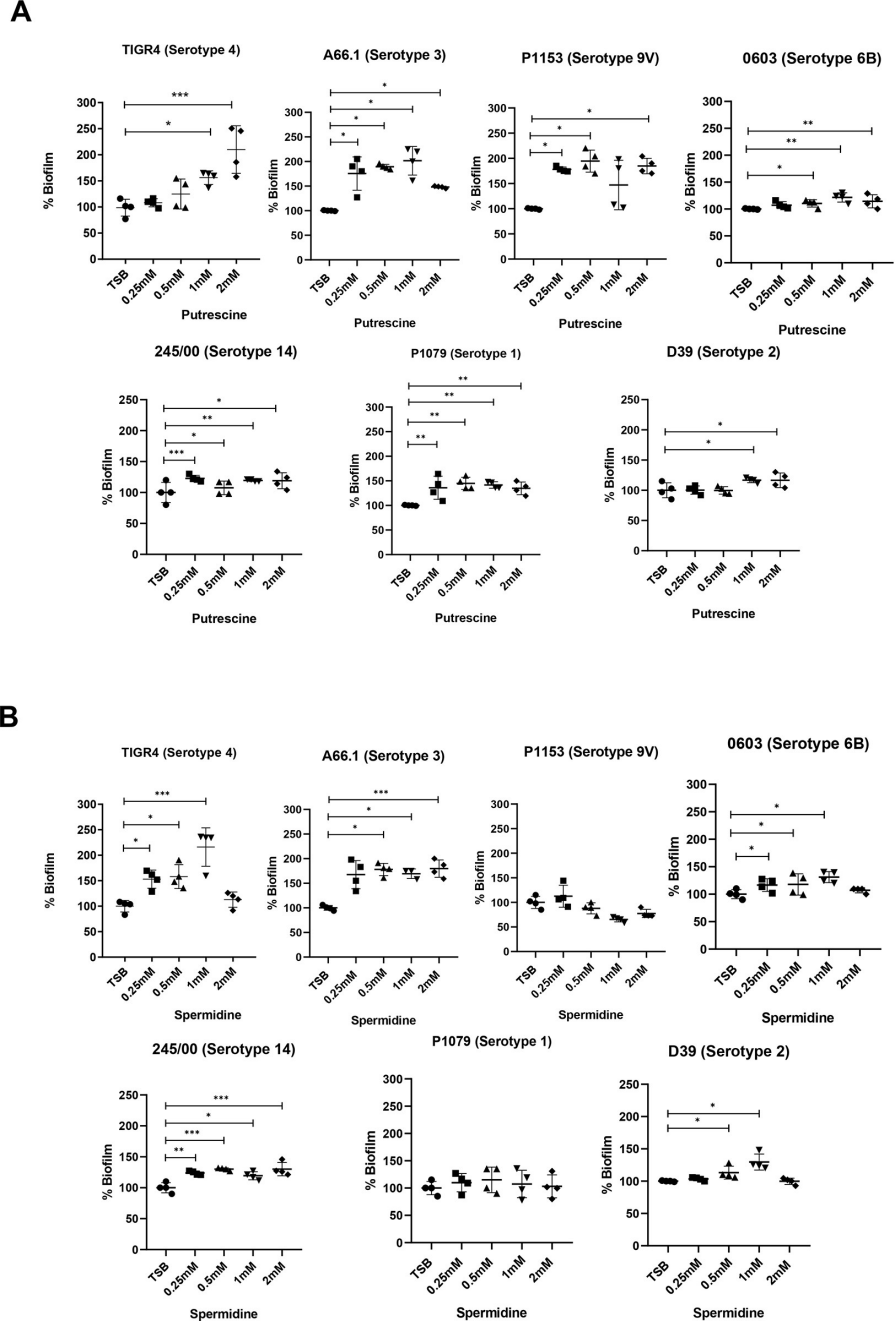
In vitro biofilm formation by *S*. *pneumoniae* with the addition of putrescine and spermidine. The biofilm was evaluated after 24 h in microplate assay with addition of 0.25 mM, 0.5 mM, 1 mM and 2 mM of **putrescine (A)** or **spermidine (B).** The biomass was evaluated by crystal violet staining at 590 nm. The absorbance from group TBS was used to calculate the percentages being considered 100%. The results expressed are representative of experiments carried out in quadruplicate. The comparison between groups was performed using the One-way ANOVA followed by Dunnet’s test, where *** = p < 0.001, *** = p<0.01 and * = p<0.05.

To investigate whether the polyamines or their transporters are important for biofilm formation by pneumococci, we added exogenous polyamines to the culture medium and evaluated the biofilm formation by different pneumococcal strains (Fig 4). Curiously, most of the strains responded to higher putrescine concentrations (1 and 2 mM) (Fig 4A), as observed for strains TIGR4, 0603 and D39, while strains P1153, P1079 and 245/00 responded to all concentrations of the molecule. The results were consistent for all strains; addition of putrescine led to an increase on biofilm formation, which varied from 115 to 130% as for strain 0603 at 0.5 to 2 mM to more than 200% as for strains TIGR4 (2 mM), A66.1 (0.5 and 1 mM), and P1153 (0,5 and 2 mM).

A similar result was observed after the addition of exogenous spermidine (Fig 4B). There was an increase in biofilm formation with 0.25, 0.5 and 1 mM of spermidine for most of the strains, with exception of P1079 and P1153 in which none of the spermidine concentrations affected biofilm formation. Strains A66.1 and D39 only start to produce more biofilm at 0.5 mM of spermidine. The reason for this observation is unknown but could be related to the capsule type and the bacterial surface charge; however more studies are needed to confirm this hypothesis.

Interestingly, the addition of 2 mM spermidine resulted in the same amount of biofilm as observed under untreated control conditions. This result was consistently repeated for most of the tested strains, with exception of the strains 245/00 and A66.1 whose biofilm formation at 2 mM was still higher than the control, similar to the other concentrations.

These results indicate that biofilm formation is affected by the polyamine availability in the medium; a richer environment provides more biofilm formation, for most of the tested conditions. For the mutant bacterium, the addition of polyamines did not affect biofilm formation, reinforcing the hypothesis that the ability to import polyamines into the cell influences the formation of biofilms by the pneumococcus (S1 Fig). A CFU count on biofilms with the addition of polyamines was also performed for strains TIGR4 and the mutant and show similar observation as for the CV staining: the addition of putrescine increased biofilm formation in almost 2 logs at concentration 0.25 and 0.5 mM, and 1 log at 1 mM. For spermidine the increase in biofilms was observed at 0.25 and 1 mM at the order of 1 log. As expected, the mutant did not respond to the addition of any polyamines as the CFU count remained similar to that of TSB alone. Furthermore, at higher concentrations of spermidine, of 2 mM, the CFU count was around 4 log lower than the control for both strains, suggesting that somehow this concentration is toxic for the pneumococcus (S1 and S2 Figs).

## Discussion

Polyamines are essential nutrients required for cells to survive. Putrescine and spermidine are the most common polyamines found in bacterial cells [10]. All cells can synthesize polyamines by metabolic pathways, but they are also capable of transporting them into the cell interior by an ABC transporter called Pot. Several studies have suggested a relationship between polyamines and/or their transporters and biofilm formation in different models [29–32].

A study by Pipkins et al. (2017) demonstrated that the absence of the *potD* caused a decrease on biofilm production when compared to the parental wild-type strain [11]. Interestingly, this was only observed in the encapsulated strain, and the opposite effect was observed in the unencapsulated strain [11]. The authors suggested that the presence of the capsule could interfere with bacterial adhesins, but this was not confirmed in the study. Our work is focused on investigating biofilm formation. For that, we have applied physiological models using epithelial cells as a substrate for the biofilm *in vitro* and have also examined biofilm formation *in vivo* using a mouse colonization experiments in addition to the abiotic microplate biofilm assay. We also provide new data showing the influence of polyamine supplementation in the culture medium on biofilm formation for different *S*. *pneumoniae* strains. Interestingly, the deletion of *pot* operon does not affect the bacterium’s ability to grow planktonically as the growth curve is similar for both strains (S3 Fig).

While *in vitro* studies from several groups have explored biofilm formation in the nasopharynx by the pneumococci [33–35], Marks et al. were the first to demonstrate that the pneumococcus is capable of forming highly structured biofilms during colonization of the upper airways in mice. This study also proposed the use of epithelial cells as a substrate *in vitro* to mimic the natural environment encountered by pneumococci during *in vivo* colonization [7]. We modified this model [6], using lung epithelial cells (A549) and our results showed that the mutant strain formed around 10 times less biofilm than the wild-type, a result that agrees with the abiotic observation. This result is interesting because it more closely mimics the natural pneumococcal niche.

Marks et al. (2012) described an *in vivo* mouse model to evaluate biofilm formation by the pneumococcus. The group identified that strains with mutations in virulence factors have less potential for biofilm formation on epithelial cells, and this is correlated with their ability to colonize the murine nasopharynx *in vivo* [6]. We used this model to evaluate the impact of *potABCD* deletion on biofilm formation *in vivo*. Our results showed that colonization was impaired in the mutant strain when compared to the wild-type strain. Interestingly, the difference between the two groups were even higher using the mouse model than previously observed in the *in vitro* models, reinforcing that more physiological models are closer to the natural environment of colonization/ biofilm development than the simpler *in vitro* models. A stable bacterial colonization after nine days of inoculation strongly indicates that the pneumococci are forming biofilms in the nasopharynx, since this is the predominant phenotype associated with persistence in that niche, as previously demonstrated by SEM [5, 6].

Finally, we wanted to investigate how the addition of polyamines in the medium affects the formation of biofilm. We tested various concentrations of exogenous putrescine and spermidine, ranging from 0.25 mM to 2 mM. The standard polyamine concentration in TSB medium is 0.4 mM. To provide context, typical levels of polyamines found in sputum samples from healthy individuals and those with cystic fibrosis are approximately 11.91 μM for putrescine and 0.88 μM for spermidine [36].

We found that supplementing the culture medium with polyamines caused an increase in biofilm formation for both polyamines transported by the Pot transporter in multiple pneumococcal strains, except in the higher concentration of spermidine, which was toxic in some cases. To understand if the observed impact of polyamines addition occurs only on biofilm cells or if it is a reflex of general bacterial growth, a biofilm assay was repeated and CFU counting was plotted considering planktonic and biofilm cells (S2 Fig). This is approach has been used by other studies [37, 38] and provides a more accurate analysis of how polyamine concentrations affect growth x biofilm formation. The result shows that the planktonic cell counts are not affected by polyamine concentrations, which is reinforced by the growth curve observed at the same figure (S2 Fig). The exception occurs for spermidine at 2 mM, which resulted in loss of planktonic bacteria, reinforcing the hypothesis that higher concentrations of spermidine are toxic for the bacterial cells.

However, the concentration of polyamines necessary to impact biofilm formation varied among the different strains, with some responding to lower doses of the molecules, while others only showed an enhancement in biofilms under higher polyamine concentrations. These differences may be due to variations in the capsular polysaccharide composition, which affect the bacterium surface charge and could attract the polyamines in variable degrees. While more studies are needed to confirm this hypothesis, the present data supports the idea that polyamines are important for biofilm formation by the pneumococcus.

Previous studies have shown that the addition of spermidine and nor-spermidine (a spermidine precursor) can regulate biofilm formation in a *Vibrio cholerae* model, our results are similar to those observations [30, 39]. Interestingly, Karatan et al. showed that adding spermidine to the medium in concentration higher than 0.5 mM inhibits the biofilm formation by *V*. *cholerae*, but no explanation was given for this fact [30]. In our experiments it was also observed as the pneumococcus produces the same amount of biofilm when the maximum concentration is added (2.0 mM) as in medium without spermidine. The CFU count reveals that this concentration could be toxic for the bacterium, since the count was diminished in this condition, even for the mutant strain, this observation could be confirmed by the CFU counts and the growth curve in the presence of spermidine (S2 Fig).

Another study investigating the relationship between polyamines and biofilms examined the behavior of a *Yersinia pestis* strain unable to synthesize putrescine. They found that the mutant produced much less biofilm than its wild-type counterpart due to a decreased concentration of intracellular putrescine [31]. Similarly, in *Pseudomonas aeruginosa*, increasing the intracellular putrescine concentration was linked to an increase in biofilm formation [40]. A recent study using *P*. *aeruginosa*, has shown that differences in the capsule charge may affect how polyamines are incorporated by the cells [41]. Our study showed that the addition of either putrescine or spermidine impacts biofilm formation *in vitro*, and the differences between different strains related to the polyamine concentrations may be explained by the differences in the capsule charges as well. Our results support the idea that the absence of the *pot* operon would reduce the amount of polyamines in the intracellular environment and thus impact bacterial ability to form biofilm.

Zhang et al. investigated polyamine transporters and biofilm in *Escherichia coli* and found that a strain modified to overexpress the PotD protein produced much more biofilm than the parental strain [42]. This work supports our results, suggesting that our observation is also true for different organisms.

Our work, however, presents some limitations, with the most notable one being the absence of a complementary strain of the mutant restoring the expression of the *potABCD* operon. We made numerous attempts to produce this strain, but all were unsuccessful. Nonetheless, this mutant has already been well characterized and used in other publications [11, 15, 43]. Our experiment involving the addition of polyamines demonstrated that the mutant did not respond to the presence of these molecules in the culture medium, maintaining the biofilm at levels identical to the medium without supplementation. This reinforces that the removal of the operon resulted only in the lack of polyamine transport into the cell. Another limitation is the absence of other pneumococcal mutant strains that lack the *pot* operon.

In conclusion, our study highlights the importance of the ABC polyamine transporter in the biofilm development by *S*. *pneumoniae*. Additionally, we demonstrate that the addition of exogenous polyamines stimulates the bacterium to produce more biofilm *in vitro*, util a limit of 2 mM, while the absence of the *pot* operon hinders biofilm formation in different models using different levels of complexity from abiotic surface to *in vivo* animal model. Interestingly, more physiological models such as mice show that the absence of the *pot* operon has a greater impact on biofilm formation. Our findings emphasize the significance of employing more physiological models in studying biofilm formation.

## Supporting information

S1 Dataset Minimal data. (XLSX)

S1 Fig *In vitro* biofilm formation by *S*. *pneumoniae* ΔPotABCD with the addition of putrescine and spermidine. The biofilm was evaluated after 24 h in microplate assay with addition of 0.25 mM, 0.5 mM, 1 mM and 2 mM of **putrescine (A)** or **spermidine (B).** The biomass was evaluated by crystal violet staining at 590 nm (left panels) and by CFU count (right panel). The absorbance from group TBS was used to calculate the percentages being considered 100%. The results expressed are representative of experiments carried out in quadruplicate. The comparison between groups was performed using the One-way ANOVA followed by Dunnet’s test.
(TIFF)

S2 Fig *In vitro* biofilm formation and planktonic growth by *S*. *pneumoniae* strain TIGR4 with the addition of putrescine and spermidine. The biofilm was evaluated after 24 h in microplate assay with addition of 0.25 mM, 0.5 mM, 1 mM and 2 mM of **putrescine (A)** or **spermidine (B).** The biofilm was evaluated by CFU count (left panels), planktonic cells (center panels) were also evaluated by CFU counting on the biofilm supernatant. The growth curve for TIGR4 was also determined at different polyamines concentration (right panels). The results expressed are representative of experiments carried out in quadruplicate. The comparison between groups was performed using the One-way ANOVA followed by Dunnet’s test *** = p < 0.001, *** = p<0.01 and * = p<0.05.
(TIFF)

S3 Fig Growth curve by *S*. *pneumoniae* strains TIGR4 and ΔPotABCD. OD600nm measurements and growth curves of S. pneumoniae strains TIGR4 and ΔPotABCD in TSB medium supplemented with 10% equine serum (Sigma-Aldrich) and incubated at 37°C under micro-aerobic conditions (5% CO_2_).
(TIFF)
