# Supplementary figures and images for: Role of the polyamine transporter PotABCD during biofilm formation by Streptococcus pneumoniae
Source: PLoS One. 2024 Aug 7;19(8):e0307573. doi: 10.1371/journal.pone.0307573 (PMC11305561; doi:10.1371/journal.pone.0307573)

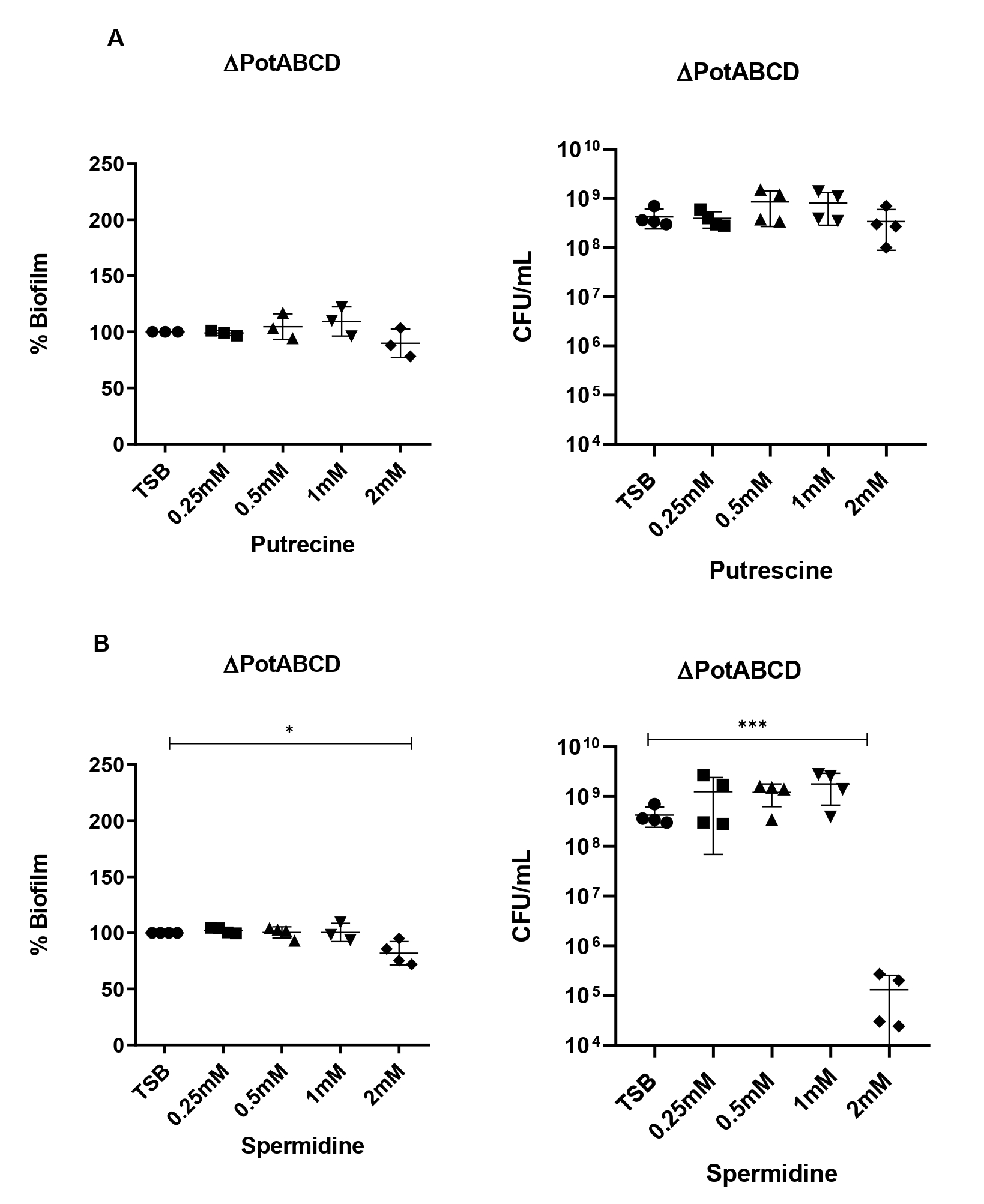

Supplement: S1 Fig — The biofilm was evaluated after 24 h in microplate assay with addition of 0.25 mM, 0.5 mM, 1 mM and 2 mM of putrescine (A) or spermidine (B). The biomass was evaluated by crystal violet staining at 590 nm (left panels) and by CFU count (right panel). The absorbance from group TBS was used to calculate the percentages being considered 100%. The results expressed are representative of experiments carried out in quadruplicate. The comparison between groups was performed using the One-way ANOVA followed by Dunnet’s test. (TIFF) [file pone.0307573.s002.tiff]

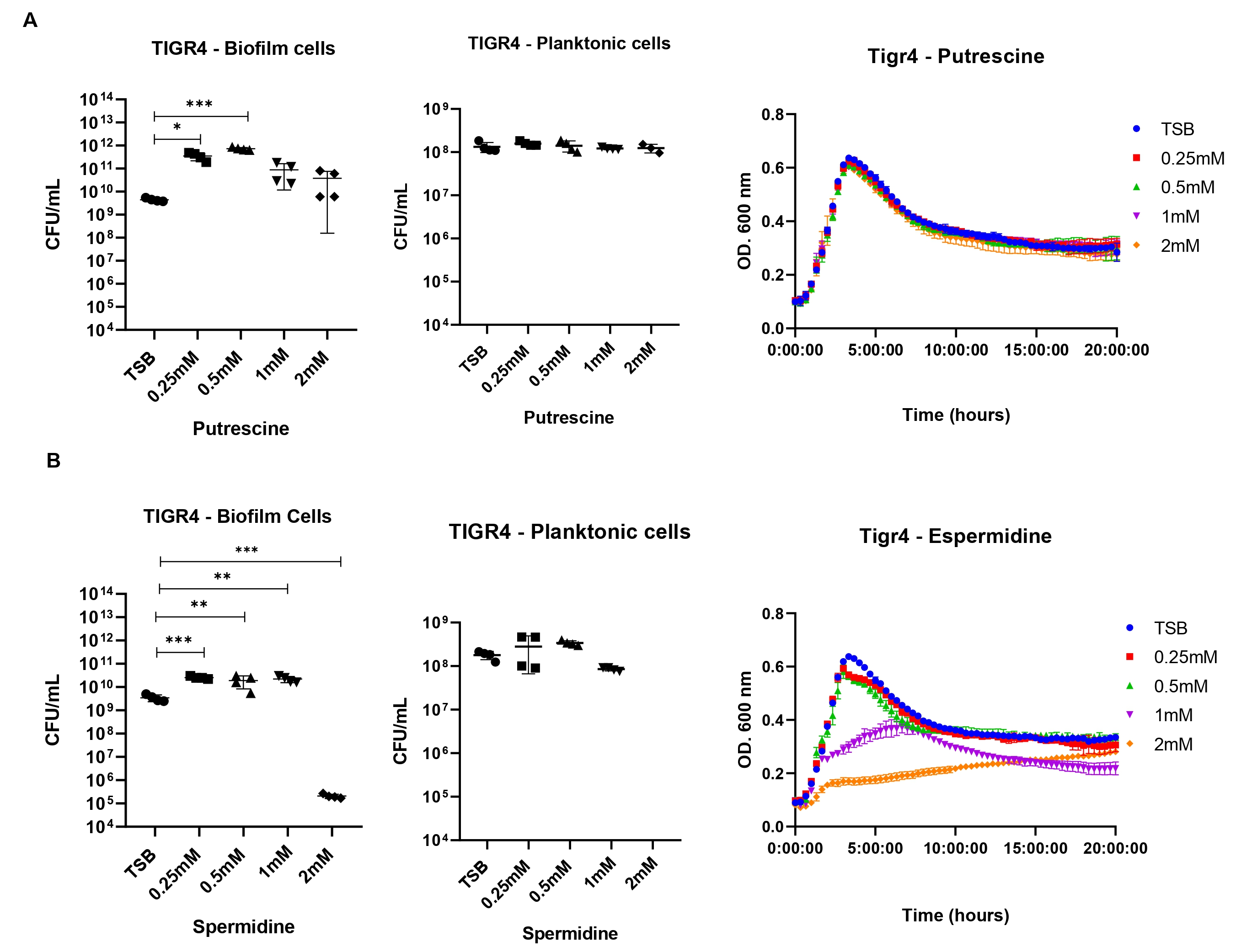

Supplement: S2 Fig — The biofilm was evaluated after 24 h in microplate assay with addition of 0.25 mM, 0.5 mM, 1 mM and 2 mM of putrescine (A) or spermidine (B). The biofilm was evaluated by CFU count (left panels), planktonic cells (center panels) were also evaluated by CFU counting on the biofilm supernatant. The growth curve for TIGR4 was also determined at different polyamines concentration (right panels). The results expressed are representative of experiments carried out in quadruplicate. The comparison between groups was performed using the One-way ANOVA followed by Dunnet’s test *** = p < 0.001, *** = p<0.01 and * = p<0.05. (TIFF) [file pone.0307573.s003.tiff]
